# Polypyridylruthenium(II) complexes exert anti-schistosome activity and inhibit parasite acetylcholinesterases

**DOI:** 10.1371/journal.pntd.0006134

**Published:** 2017-12-14

**Authors:** Madhu K. Sundaraneedi, Bemnet A. Tedla, Ramon M. Eichenberger, Luke Becker, Darren Pickering, Michael J. Smout, Siji Rajan, Phurpa Wangchuk, F. Richard Keene, Alex Loukas, J. Grant Collins, Mark S. Pearson

**Affiliations:** 1 School of Physical, Environmental and Mathematical Sciences, UNSW Canberra, Canberra, Australian Capital Territory, Australia; 2 Centre for Biodiscovery and Molecular Development of Therapeutics, Australian Institute of Tropical Health and Medicine, James Cook University, Cairns, Queensland, Australia; 3 School of Physical Sciences, University of Adelaide, Adelaide, South Australia, Australia; University of Pennsylvania, UNITED STATES

## Abstract

**Background:**

Schistosomiasis affects over 200 million people and there are concerns whether the current chemotherapeutic control strategy (periodic mass drug administration with praziquantel (PZQ)—the only licenced anti-schistosome compound) is sustainable, necessitating the development of new drugs.

**Methodology/Principal findings:**

We investigated the anti-schistosome efficacy of polypyridylruthenium(II) complexes and showed they were active against all intra-mammalian stages of *S*. *mansoni*. Two compounds, Rubb_12_-tri and Rubb_7_-tnl, which were among the most potent in their ability to kill schistosomula and adult worms and inhibit egg hatching *in vitro*, were assessed for their efficacy in a mouse model of schistosomiasis using 5 consecutive daily i.v. doses of 2 mg/kg (Rubb_12_-tri) and 10 mg/kg (Rubb_7_-tnl). Mice treated with Rubb_12_-tri showed an average 42% reduction (*P* = 0.009), over two independent trials, in adult worm burden. Liver egg burdens were not significantly decreased in either drug-treated group but ova from both of these groups showed significant decreases in hatching ability (Rubb_12_-tri—68%, Rubb_7_-tnl—56%) and were significantly morphologically altered (Rubb_12_-tri—62% abnormal, Rubb_7_-tnl—35% abnormal). We hypothesize that the drugs exerted their activity, at least partially, through inhibition of both neuronal and tegumental acetylcholinesterases (AChEs), as worms treated *in vitro* showed significant decreases in activity of these enzymes. Further, treated parasites exhibited a significantly decreased ability to uptake glucose, significantly depleted glycogen stores and withered tubercules (a site of glycogen storage), implying drug-mediated interference in this nutrient acquisition pathway.

**Conclusions/Significance:**

Our data provide compelling evidence that ruthenium complexes are effective against all intra-mammalian stages of schistosomes, including schistosomula (refractory to PZQ) and eggs (agents of disease transmissibility). Further, the results of this study suggest that schistosome AChE is a target of ruthenium drugs, a finding that can inform modification of current compounds to identify analogues which are even more effective and selective against schistosomes.

## Author summary

Schistosomiasis is a neglected tropical disease which affects over 200 million people and there is only one licensed drug, praziquantel, currently available for treatment. In a search for new drugs to control schistosomiasis, we tested the anti-schistosome efficacy of a series of ruthenium compounds and found that a number of them were able to inhibit parasite eggs from hatching and kill adult worms and praziquantel-refractory juvenile worms *in vitro*. We demonstrated that the compounds inhibit schistosome acetylcholinesterase (the enzyme that breaks down the neurotransmitter acetylcholine), which could potentially result in paralysis of the parasite, likely due to uncontrolled neuromuscular function caused by acetylcholine excess. Moreover, we showed that drug-treated worms had a significantly reduced ability to uptake exogenous glucose and markedly depleted glycogen stores, presumably through inhibition of the acetylcholinesterase-mediated glucose scavenging pathway. Lastly, we found that two of the drugs—Rubb_12_-tri and Rubb_7_-tnl—when used to treat schistosome-infected mice, were able to reduce worm burdens and significantly affect the viability of parasite eggs *in vivo*, which would have a marked impact on disease transmission. We believe that these complexes are desirable drug lead scaffolds which could be used to develop effective and selective compounds to control and treat schistosomiasis and, potentially, other parasitic diseases.

## Introduction

More than half a billion people are at risk of contracting schistosomiasis (bilharzia), a neglected tropical parasitic disease that is endemic in more than 75 countries in Africa, Asia, and South America, where over 200 million individuals are infected and approximately 280,000 die every year [1, 2]. Schistosomiasis is caused by infection with blood flukes of the genus *Schistosoma*, and disease results from the deposition of eggs in host tissues, leading to granuloma formation that can cause fibrosis, portal hypertension, and even death [3].

Despite this considerable disease burden, to date there are no vaccines against schistosomiasis [4]. Praziquantel (PZQ) remains the only effective frontline drug to treat the parasite despite its shortcomings, which include ineffectiveness against juvenile stages of the worm [5], poor efficacy in treating pre-patent infections [6], reports of reduced efficacy in field studies [7] and the inevitable risk of the development of resistant strains in response to periodic mass drug administration [8]. Since there are no alternative effective drug treatments for these parasites, new therapies are urgently required.

Among potential targets for chemotherapy are acetylcholinesterases (AChEs). These enzymes catalyze the rapid breakdown of the neurotransmitter acetylcholine (ACh) in both central and peripheral nervous systems of eukaryotic organisms, and so control neuronal function [9]. In addition to controlling cholinergic synapses, the enzyme is present in large amounts on the tegument of schistosomes [10], where it has been implicated to play a role in the regulation of host glucose uptake by the parasite by limiting the interaction of host ACh with tegumental nicotinic ACh receptors (nAChRs) [11]. These receptors are associated both spatially and temporally with surface AChE expression and are concentrated on the tegument [12], the major site of glucose uptake [13]. Evidence for this relationship is shown by the ablation of glucose uptake with a membrane-impermeable inhibitor of AChE (which has the same result as the administration of an excess of ACh) or specific agonists of nAChRs. The interaction of ACh with tegumental nAChRs is thought to decrease the amount of glucose uptake through surface glucose transporters but the specific mechanism for this is not known [11].

With respect to its termination of synaptic transmission, inhibition of AChE produces an excess accumulation of ACh and overstimulation of its receptors, causing uncoordinated neuromuscular function that often results in death due to respiratory paralysis [14]. As such, AChE inhibitors are widely used as pesticides [15] and anthelmintics [16]. Indeed, metrifonate, an organophosphorus AChE inhibitor originally used as an insecticide has also been used for the treatment of schistosomiasis until it was withdrawn from the market and further development because of off-target toxicity [17].

In addition to organophosphates, mono-nucleated chemical complexes of the transition metal ruthenium have been shown to target and inhibit enzymes such as AChE [18], and there are numerous recent studies documenting the efficacy of polypyridylruthenium(II) complexes against a variety of different microbial pathogens [19–21]. Unlike their organophosphorus counterparts, ruthenium complexes are speculated to exert their inhibitory effects through a combination of electrostatic and hydrophobic interactions at the peripheral anionic (PAS) site of AChE, which is located at the gate of the enzyme’s catalytic gorge [22], and not through direct interaction with the active site. Ruthenium complexes are thought to be less toxic to human cells than small-molecule inhibitors because of this mode of inhibition and also because the overall neutral charge in the outer membrane leaflet of eukaryotic cells [23] creates a greatly reduced capacity for electrostatic interaction with the metal compounds [24].

Herein, we demonstrate the AChE-inhibitory action of two mononuclear and a series of di-, tri- and tetra-nuclear polypyridylruthenium(II) complexes linked by the bis[4(4’-methyl-2,2’-bipyridyl)]-1,n-alkane ligand (“bb_n_”; n = 7, 10, 12 and 16) against extracts of *Schistosoma mansoni* and *Schistosoma haematobium* and both adult and juvenile *S*. *mansoni* parasites *in vitro*. We also provide evidence consistent with the capacity of these complexes to disrupt the parasite’s glucose uptake ability through the inhibition of tegumental AChE, a cholinergic pathway unique to schistosomes [11]. Finally, we show the *in vivo* efficacy of two ruthenium complexes in a mouse model of schistosomiasis, providing evidence that drugs based on these compounds could be a valuable addition to the chemotherapeutic arsenal against this debilitating disease.

## Methods

### Nomenclature and preparation of ruthenium complexes

**Fig 1:**
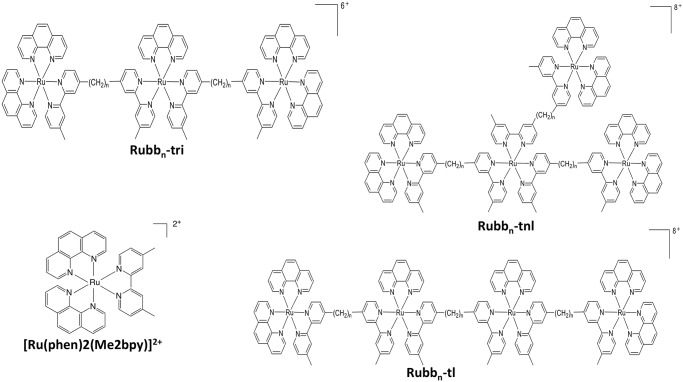
The kinetically inert tri-nuclear (Rubb_n_-tri), linear tetra-nuclear Rubb_n_-tl and non-linear tetra-nuclear Rubb_n_-tnl) ruthenium(II) complexes.

[Ru(phen)_2_(Me_2_bpy)]^2+^ and the mononuclear (Rubb_n_-mono), dinuclear (Rubb_n_-di), trinuclear (Rubb_n_-tri), tetranuclear linear (Rubb_n_-tl) and tetranuclear non-linear (Rubb_n_-tnl) polypyridylruthenium(II) complexes (Fig 1) were synthesised using the appropriate bis[4′-(4-methyl-2,2′-bipyridyl)]-1,n-alkane bridging ligand (bb_n_) as previously described [19]. Compounds were dissolved in H_2_O at stock concentrations of 1 mM.

### Parasites and extracts

*S*. *mansoni* cercariae were shed from infected *Biomphalaria glabrata* snails (Biomedical Research Facility, MD, USA) by exposure to light at 28°C for 2 hours. Cercariae were used to infect 6–8 week old male BALB/c mice (Animal Resources Centre, WA, Australia) by tail penetration (180 *S*. *mansoni* cercariae/mouse) and adults were harvested by vascular perfusion at 7 weeks post-infection [25]. *S*. *mansoni* eggs were purified from infected mouse livers as previously described [26] and were used in the xWORM egg hatching assay or to make soluble egg antigen (SEA) [27]. For experiments involving schistosomula, *S*. *mansoni* cercariae were mechanically transformed as previously described [27]. To make PBS-soluble extracts, *S*. *mansoni* adult worms were homogenized in PBS (50 μl/worm pair) at 4°C using a TissueLyser II (Qiagen) and the supernatant collected by centrifugation at 15,000 *g* for 60 mins at 4°C. Triton X-100-soluble extracts of *S*. *mansoni* and *S*. *haematobium* were made in the same way except worms were lysed in buffer containing 1% Triton X-100, 40 mM Tris-HCl, pH 7.4. Protein concentration was determined using the Pierce BCA Protein Assay kit (Thermofisher), aliquoted and stored at -80°C until use.

### Enzyme activity in parasite extracts and inhibition assays

AChE, nucleotide pyrophosphatase-phosphodiesterase 5 (SmNPP-5) and alkaline phosphatase (AP) activity in Triton X-100-soluble adult worm extracts and AChE activity in SEA were determined in a Polarstar Omega microplate reader (200 μl final volume in 96-well plates). AChE activity was determined using the Ellman method [28]; extracts were serially diluted (20–5 μg) in AChE assay buffer (0.1 M sodium phosphate, pH 7.4), 2 mM acetylthiocholine (AcSCh) and 0.5 mM 5,5’-dithio-bis(2-nitrobenzoic acid) (DTNB) were added and absorbance was measured at 405 nm every 10 min for 5 h at 37°C. Specific activity was calculated using the initial velocity of the reaction. For AChE inhibition assays, parasite extracts equal to a specific activity of 0.55 nmol/min/well were diluted in AChE assay buffer to a final volume of 170 μl and pre-incubated with ruthenium complexes (10 nM– 100 μM) for 20 min at RT. AcSCh and DTNB were added at 2 mM and 0.5 mM, respectively and absorbance was measured at 405 nm every 10 min for 5 h at 37°C. SmNPP-5 activity [29] was measured by serially diluting extracts in SmNPP-5 assay buffer (50 mM Tris-HCl, pH 8.9, 120 mM NaCl, 5 mM KCl, 60 mM glucose), adding 0.5 mM p-nitrophenyl thymidine 5′-monophosphate (p-Nph-5’-TMP) and reading the absorbance (405 nm) every 10 min for 5 h at 37°C. Specific activity was calculated using the initial velocity of the reaction. For SmNPP-5 inhibition assays, parasite extract equal to 32 nmol/min/well was diluted in SmNPP-5 assay buffer to a final volume of 180 μl and pre-incubated with ruthenium complexes (10 nM– 100 μM) for 20 min at RT. Substrate (p-Nph-5’-TMP) was added to 0.5 mM and absorbance was measured at 405 nm every 10 min for 5 h at 37°C. AP activity [30] was measured by serially diluting extracts in AP assay buffer (0.1 M glycine, pH 10.4, 1 mM MgCl_2_, 1 mM ZnCl_2_), adding 2 mM p-nitrophenyl phosphate (pNPP) and measuring absorbance (λ = 405 nm) every 2 min for 1 h at 37°C. Specific activity was calculated using the initial velocity of the reaction. For AP inhibition assays, parasite extract equal to 1 nmol/min/well was diluted in AP assay buffer to a final volume of 180 μl and pre-incubated with ruthenium complexes (10 nM– 100 μM) for 20 min at RT. Substrate (pNPP) was added to 2 mM and absorbance was measured at 405 nm every 2 min for 1 h at 37°C. For all assays, inhibition for each sample was calculated relative to the negative control (reactions without ruthenium complexes) and reactions were performed in duplicate with data presented as the average ± SEM.

### Effect of ruthenium complexes against larval *S*. *mansoni* parasites

Newly-transformed schistosomula (100 parasites) were cultured (37°C, 5% CO_2_) in 200 μl of Basch medium supplemented with 4× antibiotic/antimycotic (AA—200 units/ml penicillin, 200 μg/ml streptomycin and 0.5 μg/ml amphotericin B) in a 96 well plate in the presence of a series of serially-diluted ruthenium complexes (100 μM– 10 nM). After 48 h, parasite viability was assessed microscopically by trypan blue exclusion staining as previously described [31]. Six of the most effective compounds were tested again for their larvacidal efficacy (100 schistosomula per treatment); this time at concentrations of 200, 100, 50, 25, 12.5 and 6.25 μM. Experiments were performed in duplicate with IC_50_ data presented as the average ± SE.

### Effect of ruthenium complexes against adult *S*. *mansoni* parasites

Five pairs of adult worms were cultured in 2 ml of Basch medium supplemented with 4x AA in a 24 well plate in the presence of ruthenium complexes (50 μM). Control worms were treated with an equal amount of H_2_O. Worms were cultured at 37°C and 5% CO_2_ for 7 days, monitored every 24 h for motility by microscopic examination and considered dead if no movement was seen. The most effective ruthenium complexes (5 compounds) were tested in duplicate (five pairs of worms each) at 10, 50 and 100 μM. Data is presented as the average of each duplicate experiment ± SE.

### Effect of ruthenium complexes on *S*. *mansoni* egg hatching and development

Egg hatching was evaluated by the xWORM egg hatching assay [32]. Ova (5,000 per well, 200 μl reaction volume) were incubated in 0.1x PBS, pH 7.2, containing ruthenium complexes (50 μM) and induced to hatch under bright light at RT for 16 h. The motility of the miracidia released from the hatched eggs was monitored every 15 s and the motility index was calculated as described [32]. Experiments were performed in triplicate with control reactions containing no ruthenium compounds. To investigate the effects of ruthenium complexes on egg development, triplicate sets of five pairs of adult *S*. *mansoni* worms were cultured in Basch media with or without 5 μM Rubb_12_-tri, for 72 h. The eggs released into the media were counted and misshapen, malformed or immature eggs [33] were scored as “abnormally developed”. Egg hatching and morphology data are presented as the average of each triplicate experiment ± SE.

### Assessment of enzyme inhibitory effects induced by treatment of worms with ruthenium complexes

Freshly perfused worms were cultured in the presence of sub-lethal concentrations (5 μM) of Rubb_12_-tri or Rubb_16_-tnl—two ruthenium compounds determined to be most effective in terms of their combined activity against schistosomula, adult worms and eggs—in Basch medium at 37°C and 5% CO_2_. To measure surface AChE or AP activity, 5 pairs of worms (preliminary experiments by us showed this number of parasites was sufficient to accurately measure surface enzyme activity) were transferred to either AChE assay buffer (0.1 M sodium phosphate, pH 7.4, 2 mM AcSCh, 0.5 mM DTNB) or AP assay buffer (0.1 M glycine, pH 10.4, 1 mM MgCl_2_, 1 mM ZnCl_2_, 2mM p-nitrophenyl phosphate). Surface enzyme activities were quantified by measuring the absorbance (λ = 405 nm) after incubation for 60 min (AChE) or 30 min (AP). Activity was measured from triplicate sets of parasites (5 pairs of worms) and each assay was technically replicated three times. For each enzyme assay, activities of drug-treated parasites were expressed relative to worms cultured without ruthenium complexes (negative controls). To measure somatic AChE activity, PBS-soluble extracts were made from worms used for surface AChE activity (triplicate sets of five pairs of worms) and then assayed in triplicate as described above. Data is presented as the average of each triplicate biological and technical experiment ± SE.

### Effect of glucose uptake and glycogen storage on worms treated with ruthenium complexes

Five pairs of freshly-perfused worms were cultured in the presence of sub-lethal concentrations (5 μM) of Rubb_12_-tri or Rubb_16_-tnl in DMEM (1000 mg/l glucose). Media (20 μl) from each experiment was collected after 24 h and the amount of glucose was quantified using a colorimetric glucose assay kit (Sigma) according to the manufacturer’s instructions. Media was collected from triplicate sets of parasites (five pairs of worms) and each assay was replicated 3 times. Glucose levels were expressed relative to media collected from worms which received no drug (negative control). Data is presented as the average of each triplicate biological and technical experiment ± SE. To measure the glycogen content of worms treated with ruthenium drugs, Triton X-100-soluble extracts were made and assayed for glycogen in a modified procedure described by Gomez- Lechon et al. [34]. Briefly, 0.2 M sodium acetate, pH 4.8, was added to 30 μg parasite extract and 50 μl glucoamylase (10 U/ml) to make a reaction volume of 150 μl. The mixture was incubated at 40°C for 2 h with shaking at 100 rpm, 40 μl added to a new microplate with 10 μl 0.25 M NaOH and the amount of glucose quantified using the colorimetric glucose assay kit. Extracts were made from triplicate sets of parasites (five pairs of worms) and assays were performed three times. Data is presented as the average of each triplicate biological and technical experiment ± SE.

### Scanning electron microscopy

Control parasites and worms treated with 5 μM Rubb_12_-tri were prepared for scanning electron microscopy by fixation in 3% glutaraldehyde followed by successive dehydration for 15 mins each in a graded ethanol series (100%, 90%, 80%, 70%, 60%, 50%), 1:1 ethanol:hexamethyldisilizane (HMDS) and, finally, 100% HMDS. Dehydrated worms were covered and left overnight in a fume hood to allow the HMDS to evaporate then mounted on an aluminium stub, sputtered with gold and visualized using a JEOL JSM scanning electron microscope operating at 10 kV. Images were acquired digitally using Semaphore software.

### Cytotoxicity assays

Cytotoxicity assays were performed using the mitochondrial-dependent reduction of 3-(3,4-dimethylthiazol-2yl)-5-diphenyl tetrazolium bromide (MTT) to formazan as described by Pandrala et al. [35]. The human bile duct cell line H69 was cultured in 96-well microtiter plates containing 0.1 ml of growth factor-supplemented media (DMEM/F12 with high glucose (4 mg/ml), 10% FCS, 1×AA, 25 μg/ml adenine, 5 μg/ml insulin, 1 μg/ml epinephrine, 8.3 μg/ml holo-transferrin, 0.62 μg/ml hydrocortisone, 13.6 ng/ml triiodo-1-thyronine (T3) and 10 ng/ml epithelial growth factor (EGF)) [36] to a cell density of 5,000 per well at 37°C in 5% CO_2_. Cell viability was assessed after continuous exposure to a concentration series (50, 25, 10, 5, 1, 0.5 and 0.1 μM) of Rubb_12_-tri, Rubb_16_-tnl, PZQ or dichlorvos—a metabolite of the anti-schistosome AChE inhibitor metrifonate—for 72 h. The amount of reduced MTT to formazan within the cells was quantified by measuring the absorbance at λ = 550 nm. Data are the average of six replicate experiments ± SE.

### Tolerability study

In order to determine the maximum tolerated dose of Rubb_12_-tri and Rubb_7_-tnl to be administered to mice infected with *S*. *mansoni*, five intravenous (i.v.) doses (tail vein) of Rubb_12_-tri and Rubb_7_-tnl were given to groups of three male BALB/c mice (6–8 weeks) daily for five consecutive days. The doses ranged from 0.25 to 10 mg/kg in PBS and were administered in a volume of 30 μl. Animals were closely monitored for adverse clinical signs throughout the study and mice showing adverse effects were euthanized using CO_2_ asphyxiation. The highest dose that did not cause any adverse clinical signs was considered to be the maximum tolerated dose (MTD) for five consecutive daily doses.

### *In vivo* efficacy of ruthenium complexes

Groups of 8 male BALB/c mice (6–8 weeks) were infected with *S*. *mansoni* cercariae as described above. At 35 days post-infection, groups were given five consecutive daily i.v. doses (tail vein—30 μl) of the MTD of either Rubb_12_-tri (2 mg/kg in PBS) or Rubb_7_-tnl (10 mg/kg in PBS). PBS was similarly administered to the control group. Two independent trials were performed. Parasites were harvested by vascular perfusion at 49 days post-infection and the average worm burden per mouse for each group of mice (trial 1 PBS control—n = 8 mice, trial 1 Rubb_12_-tri-treated—n = 8 mice, trial 1 Rubb_7_-tnl-treated—n = 6 mice, trial 2 PBS control—n = 7 mice, trial 2 Rubb_12_-tri-treated—n = 7 mice, trial 2 Rubb_7_-tnl-treated—n = 8 mice) was calculated. Data is presented as a combination of the two independent trials ± SE. Livers from each group (trial 1 PBS control—n = 8 mice, trial 1 Rubb_12_-tri-treated—n = 8 mice, trial 1 Rubb_7_-tnl-treated—n = 6 mice, trial 2 PBS control—n = 7 mice, trial 2 Rubb_12_-tri-treated—n = 7 mice, trial 2 Rubb_7_-tnl-treated—n = 8 mice) were collected, halved and weighed, with one half digested with 5% KOH to determine liver eggs per gram of tissue (epg) as previously described [37]. The other half of each liver was pooled according to group, homogenized in H_2_O and placed in identical foil-covered volumetric flasks under bright light to hatch eggs released from the livers. After 1 h, the number of miracidia in 10 x 50 μl aliquots of H_2_O (sampled from the extreme top of each flask) were counted. The amount of eggs in each flask at the start of the hatching experiment was determined by liver epg calculations, allowing the egg hatching index of each group to be calculated by expressing the hatched eggs (miracidia) as a percentage of the total eggs. Data presented is for trial 1 only and represents the average of ten counts ± SE. To assess fitness of parasites recovered from mice treated with ruthenium complexes compared to controls, worms recovered from each group were pooled and five pairs were assayed for surface enzyme activity (*Sm*-AChE, SmNPP-5 and *Sm*-AP) or glucose uptake ability as described above. Somatic *Sm*-AChE activity was determined from homogenates made from the worms assayed for surface *Sm*-AChE activity, also as described above. Each assay was technically replicated three times and data is presented as the average of triplicate technical replicates of both trials combined ± SE. To compare eggs released from parasites recovered from mice treated with ruthenium complexes and controls, triplicate sets of worms (five pairs) from each pool were incubated in Basch media at 37°C in 5% CO_2_ for 72 h. The number of eggs released were counted and scored on the basis of development and morphology [33] by visualization under a FITC filter on a Zeiss AxioImager-M1 fluorescent microscope. Data presented is for trial 2 and is the average of triplicate experiments ± SE.

### Statistical analyses

Statistical analyses were performed using Graphpad Prism 7. Inhibition curves and IC_50_ values were generated using sigmoidal dose-response (variable slope) with a non-linear fit model. One-way ANOVA with Dunn’s multiple comparison was used to determine significance (*p*), which was set at 0.05. In the case where only two groups were compared, student’s *t* test was used.

### Animal ethics approval

The James Cook University (JCU) animal ethics committee approved all experimental work involving animals (ethics approval number A2271). Mice were raised in cages in the JCU quarantine facility for the duration of the experiments in compliance with the 2007 Australian Code of Practice for the Care and use of Animals for Scientific Purposes and the 2001 Queensland Animal Care and Protection Act. All reasonable efforts were made to minimise animal suffering.

## Results

### Inhibition of AChE in schistosome extracts by ruthenium complexes

**Fig 2:**
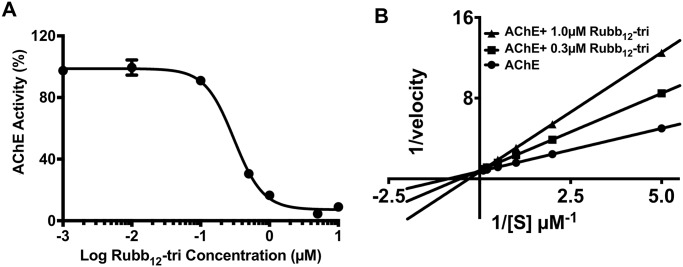
Effect of ruthenium complexes on AChE activity in adult *S*. *mansoni* extracts. Concentration-dependent inhibition of AChE activity in *S*. *mansoni* adult extracts when treated with Rubb_12_-tri, a representative member of the ruthenium complexes tested, as determined by Ellman assay. (A) Dose-response curve of Rubb_12_-tri. (B) Lineweaver-Burk inhibition plot of AChE activity in *S*. *mansoni* adult extracts in the presence of Rubb_12_-tri. Data represent the average of triplicate experiments ± SE.

A series of ruthenium complexes of different nuclearity (mono-, di-, tri- and tetra-linear and tetra-nonlinear) and with different chain lengths in the linking ligand (bb_7_, bb_10_, bb_12_, bb_16_) were screened (1 μM) for AChE inhibitory activity in Triton X-100-soluble extracts from adult *S*. *mansoni* and *S*. *haematobium* and *S*. *mansoni* soluble egg antigen (SEA) (Table 1). In *S*. *mansoni* extracts, all tri- and tetra-nuclear complexes inhibited AChE activity by 70–90% and 7 of the 13 compounds had IC_50_ values ≤ 1 μM. A dose-response curve and Lineweaver-Burk plot is shown for the most potent of these complexes (Rubb_12_-tri, IC_50_ = 0.3 μM) (Fig 2).

**Table 1:** Inhibition of acetylcholinesterase (AChE) activity in adult *S*. *mansoni* and *S*. *haematobium* Triton X-100-soluble extracts and *S*. *mansoni* soluble egg extract (SEA) by a series of ruthenium complexes.

ta	*S*. *mansoni* extract		*S*. *haematobium* extract		*S*. *mansoni* SEA	
	AChE Inhibition (%)^a^^,^ ^b^	IC_50_ (μM)^b^	AChE Inhibition (%)^a^^,^ ^b^	IC_50_ (μM)^b^	AChE Inhibition (%)^a^^,^ ^b^	IC_50_ (μM)^b^
Ru(phen)_2_(Me_2_bpy)	9.3 ± 1.8	67.0 ± 11.6	13.3 ± 1.8	90.2 ± 1.2	66.5 ± 5.8	ND
Rubb_12_-mono	11.1 ± 0.8	18.9 ± 2.8	52.2 ± 0.5	1.1 ± 0.1	73.5 ± 4.9	ND
Rubb_12_-di	73.9 ± 0.5	1.6 ± 0.3	20.9 ± 0.2	16.8 ± 0.4	94.2 ± 0.8	ND
Rubb_7_-tri	84.3 ± 4.5	0.4 ± 0.0	37.2 ± 0.3	0.4 ± 0.0	77.5 ± 0.6	ND
Rubb_10_-tri	78.7 ± 0.7	0.5 ± 0.0	73.6 ± 2.0	3.0 ± 0.0	91.3 ± 0.3	ND
Rubb_12_-tri	89.4 ± 0.4	0.3 ± 0.0	87.6 ± 0.4	0.7 ± 0.0	96.3 ± 1.4	0.2 ± 0.0
Rubb_16_-tri	46.8 ± 1.1	1.9 ± 0.0	31.6 ± 0.5	18.0 ± 0.1	89.7 ± 0.1	ND
Rubb_7_-tl	73.4 ± 1.5	1.0 ± 0.0	24.8 ± 0.3	36.4 ± 4.4	78.7 ± 8.0	ND
Rubb_12_-tl	89.9 ± 1.6	0.3 ± 0.0	55.4 ± 2.6	1.0 ± 0.1	97.2 ± 0.6	0.2 ± 0.0
Rubb_16_-tl	76.3 ± 1.6	2.4 ± 0.1	59.3 ± 0.1	1.9 ± 0.1	90.8 ± 0.2	ND
Rubb_7_-tnl	84.0 ± 0.6	0.9 ± 0.1	19.5 ± 0.3	ND	98.1 ± 0.3	ND
Rubb_12_-tnl	80.0 ± 0.1	0.4 ± 0.0	75.9 ± 2.5	3.2 ± 0.0	92.7 ± 0.3	0.3 ± 0.0
Rubb_16_-tnl	73.0 ± 0.8	2.3 ± 0.1	51.1 ± 6.0	ND	89.6 ± 0.1	ND

^a^inhibition of *Sm*-AChE activity assessed at 1μM.

^b^data represents the mean of duplicate experiments ± SE

Interestingly, a different pattern of inhibition by the ruthenium complexes was observed against AChE activity in *S*. *haematobium* extracts with the IC_50_ values being considerably more varied than was observed for AChE activity in *S*. *mansoni* adult extracts, and not all compounds showed correlated potency between the two species. In addition, there was more variability among the tri- and tetra-nuclear complexes with the more lipophilic complexes (e.g. Rubb_10_-tri and Rubb_12_-tri) having stronger inhibitory activity. Rubb_12_-mono, Rubb_12_-tri and Rubb_12_-tnl showed greater activity and the IC_50_ values of these complexes were less than 1 μM. Overall, ruthenium compounds displayed a similar pattern of inhibition against AChE activity in *S*. *mansoni* egg versus adult extracts, although most complexes showed a stronger inhibitory capacity towards AChE activity in SEA with three compounds (Rubb_12_-tri, Rubb_12_-tl and Rubb_7_-tnl) achieving >95% inhibition.

In order to examine the selectivity for AChE, the series of ruthenium complexes was screened (10 μM) against *S*. *mansoni* extract for inhibition of two major tegumental enzymes—the phosphodiesterase SmNPP-5 and alkaline phosphatase (AP). None of the compounds strongly inhibited activity of either enzyme at 10 μM, a tenfold higher concentration than was used for the AChE inhibition assays (S1 Table).

### *In vitro* effect of ruthenium complexes on larval *S*. *mansoni* parasites

The entire series of Ru complexes were screened for their larvacidal activity against *S*. *mansoni* schistosomula with IC_50_ values calculated for the most effective compounds in a separate experiment. (Table 2).

**Table 2:** Potency of selected ruthenium complexes against *S*. *mansoni* schistosomula after 48 h treatment.

Compound	IC_50_ (μM)^1^
Rubb_12_-tri	45.1 ± 4.8
Rubb_12_-tl	68.5 ± 3.6
Rubb_12_-tnl	42.8 ± 1.2
Rubb_7_-tl	81.3 ± 0.6
Rubb_7_-tnl	30.3 ± 2.0
Rubb_16_-tnl	27.3 ± 0.4

^1^data represents the mean of duplicate experiments ± SE

### *In vitro* effect of ruthenium complexes on adult *S*. *mansoni* parasites

**Fig 3:**
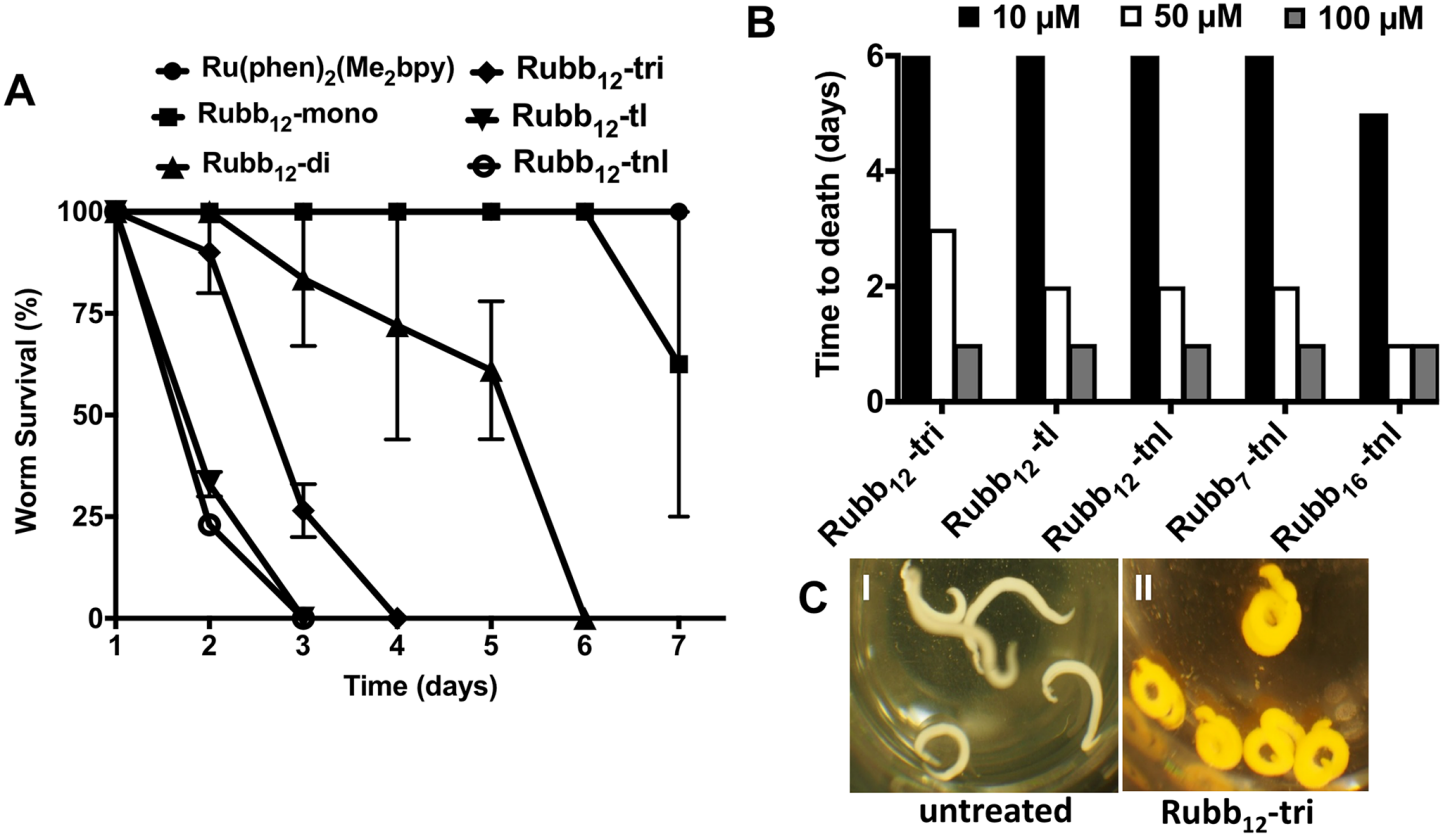
Activity of ruthenium complexes against adult *S*. *mansoni* worms. (A) Selective representation of survival of parasites (cultured in Basch media) after treatment with Ru complexes (50 μM). Data represents the average of duplicate experiments ± SE. (B) Survival of parasites (cultured in Basch media) after treatment with various concentrations of the most potent ruthenium complexes determined from the screening experiment. Data represents the average of duplicate experiments ± SE. (C) Alteration in general morphology of adult *S*. *mansoni* worms caused by ruthenium complexes. I: control parasites; II: parasites treated with Rubb_12_-tri.

*S*. *mansoni* worms were cultured in the presence of 50 μM of each ruthenium complex to investigate their effectiveness in killing adult parasites, which was assessed by lack of motility. The effects of selected compounds on worm survival is shown in Fig 3A. Similar to the enzyme inhibition in parasite extracts, the tri-and tetra-nuclear complexes were the most effective compared to the mono- and di-nuclear complexes. The killing ability increased with the increasing number of ruthenium centres in the complex, and the tetra-nonlinear complexes were more active in comparison with their linear counterparts. As with the schistosomula killing experiment, the most effective compounds (five) were tested again, this time at concentrations of 100 μM, 50 μM and 10 μM (Fig 3B). All tri-and tetra-nuclear complexes (10 μM) killed 100% of the parasites in six days. Treatment with the ruthenium complexes induced significant changes in the gross morphology of the parasites (Fig 3C). In particular, a tight coiling of the treated worms was observed.

### *In vitro* effect of ruthenium complexes on *S*. *mansoni* egg hatching and development

**Fig 4:**
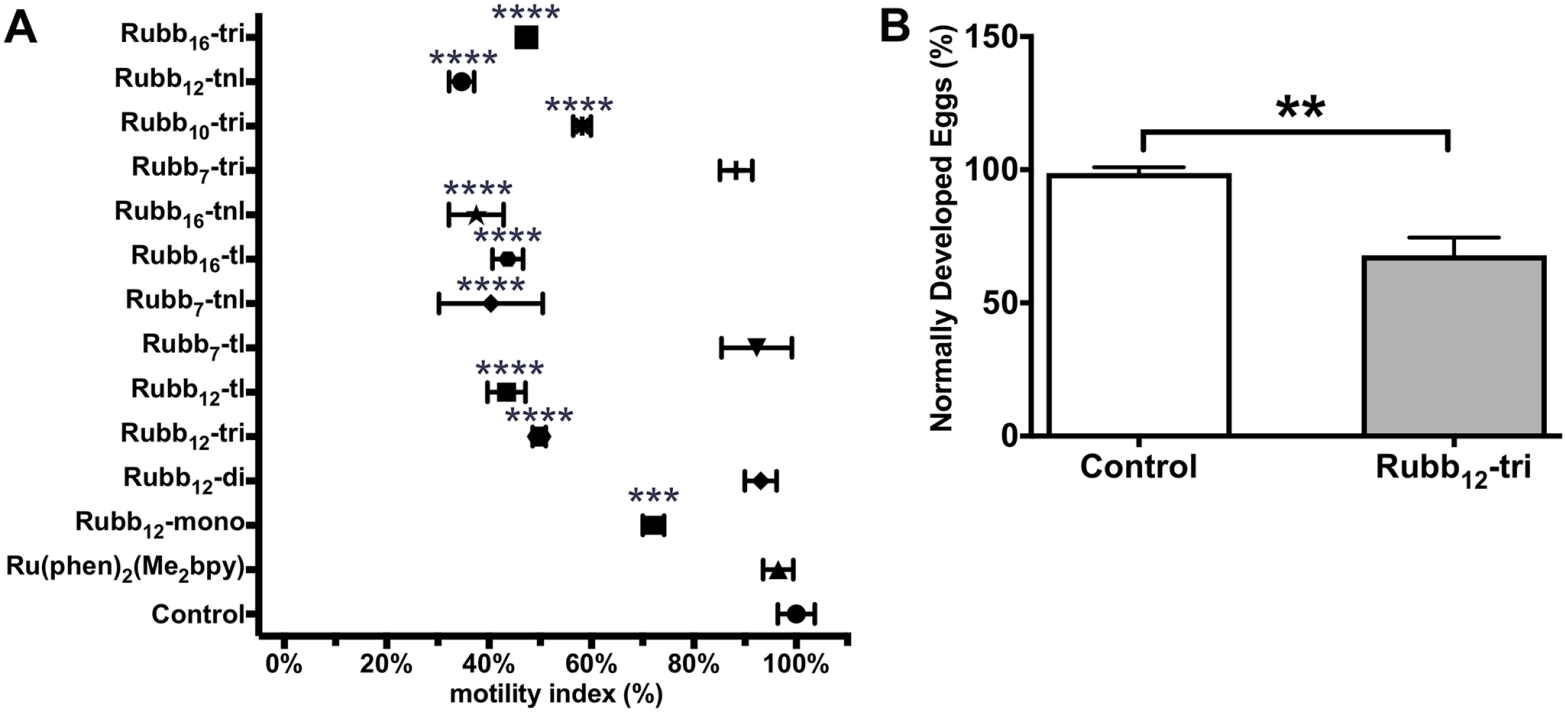
Inhibition of *S*. *mansoni* egg hatching and effect on egg development by ruthenium complexes. (A) Graph representing the percentage of *S*. *mansoni* eggs hatched (motility index) in the presence of various ruthenium complexes (50 μM) as determined by the x-WORM motility assay. Data represents the average of triplicate experiments ± SE. (B) Triplicate sets of five pairs of adult *S*. *mansoni* worms were cultured in Basch media with or without 5 μM Rubb_12_-tri for 72 h. The eggs released into the media were counted and those that were misshapen or immature were scored as “abnormally developed”. Graph shows the difference in percentage of normally developed eggs between treated and control groups and data represents the average of triplicate experiments ± SE. Differences in egg hatching were measured by ANOVA and differences in egg development by t test. *P ≤ 0.05, **P ≤ 0.01, ***P ≤ 0.001,****P ≤ 0.0001.

*S*. *mansoni* egg hatching in the presence (50 μM) of ruthenium complexes was investigated by measuring the motility index of hatched miracidia from eggs using the xWORM assay (Fig 4A). Significant reduction in hatching/motility was observed for 9/13 compounds tested. Rubb_12_-tnl was the most effective, reducing the motility index by 67% (*P* < 0.0001). To analyze the effect of ruthenium complexes on egg development, the eggs released from worms incubated for 3 days in the presence of 5 μM Rubb_12_-tri were scored for morphology. Eggs released from treated worms were abnormally developed (immature or misshapen) compared to controls (Fig 4B; *P* < 0.01).

### Mechanism of anti-schistosome action of Rubb_12_-tri and Rubb_16_-tnl

**Fig 5:**
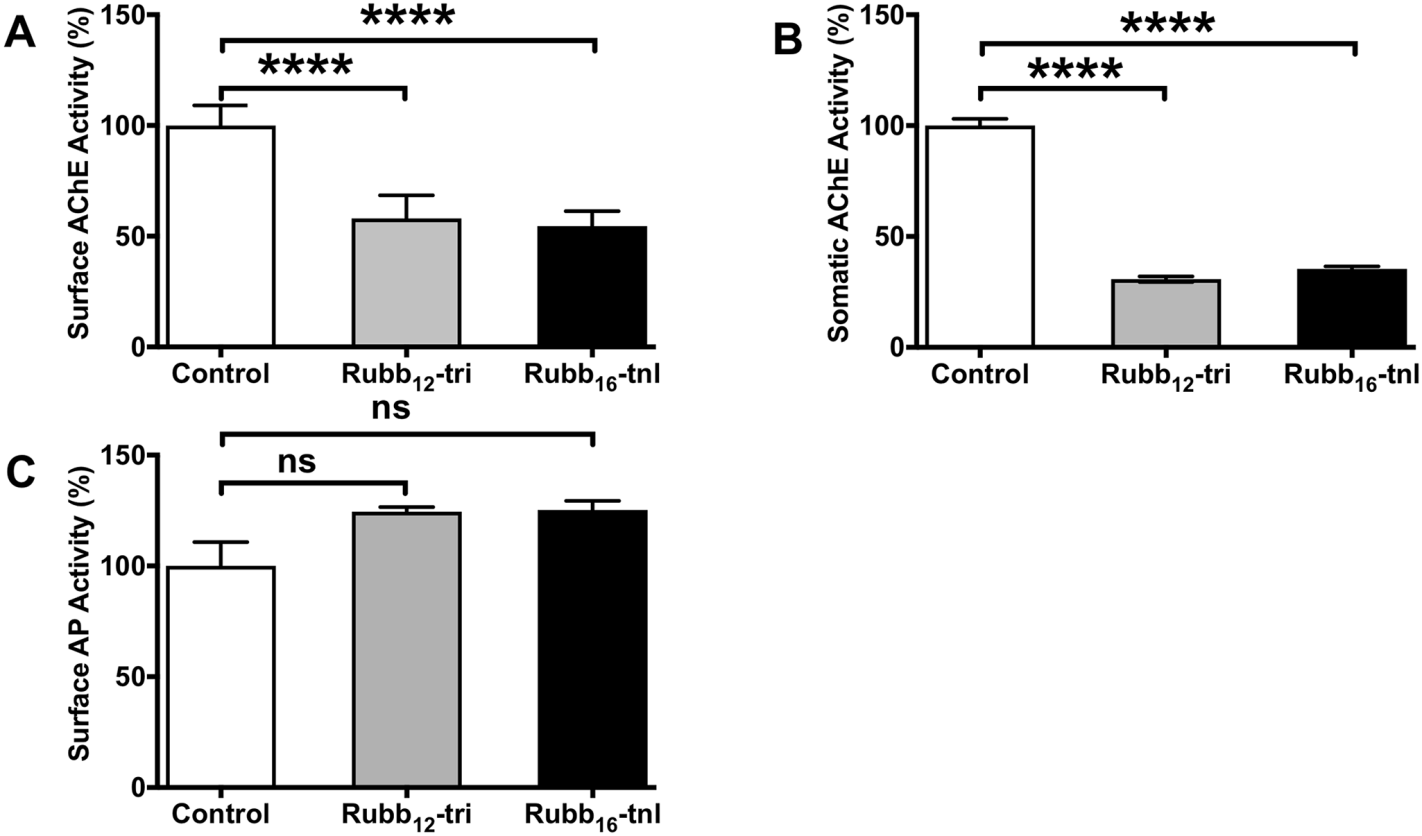
Action of Rubb_12_-tri and Rubb_16_-tnl on adult *S*. *mansoni* AChE and AP activity. Five pairs of worms were cultured in Basch media for 24 h in the presence of a sub-lethal dose (5 μM) of Rubb_12_-tri or Rubb_16_-tnl and then incubated in AChE assay buffer or AP assay buffer. PBS extracts were then made from equal amounts of control and treated worms and 30 μg of each extract was used to determine somatic AChE activity by the Ellman method. (A) surface AChE (B) somatic AChE and (C) surface AP activity of control worms and worms treated with Rubb_12_-tri or Rubb_16_-tnl. For all assays, data are the average of triplicate biological and technical experiments ± SE. Differences were measured by ANOVA. *P ≤ 0.05, **P ≤ 0.01, ***P ≤ 0.001, **** *P* ≤ 0.0001.

Adult worms were cultured in the presence of sub-lethal concentrations (5 μM) of Rubb_12_-tri or Rubb_16_-tnl—the two ruthenium compounds deemed to be most effective at killing adult parasites—for 24 h and then examined for changes in surface and somatic AChE activity and glucose uptake (given this pathway can be ablated by an organophosphorus AChE inhibitor). Treated worms showed significantly decreased levels of surface and somatic AChE activity in the presence of each complex (Fig 5A and 5B) however, consistent with enzyme inhibition experiments using parasite extracts, AP activity was not significantly affected (Fig 5C).

**Fig 6:**
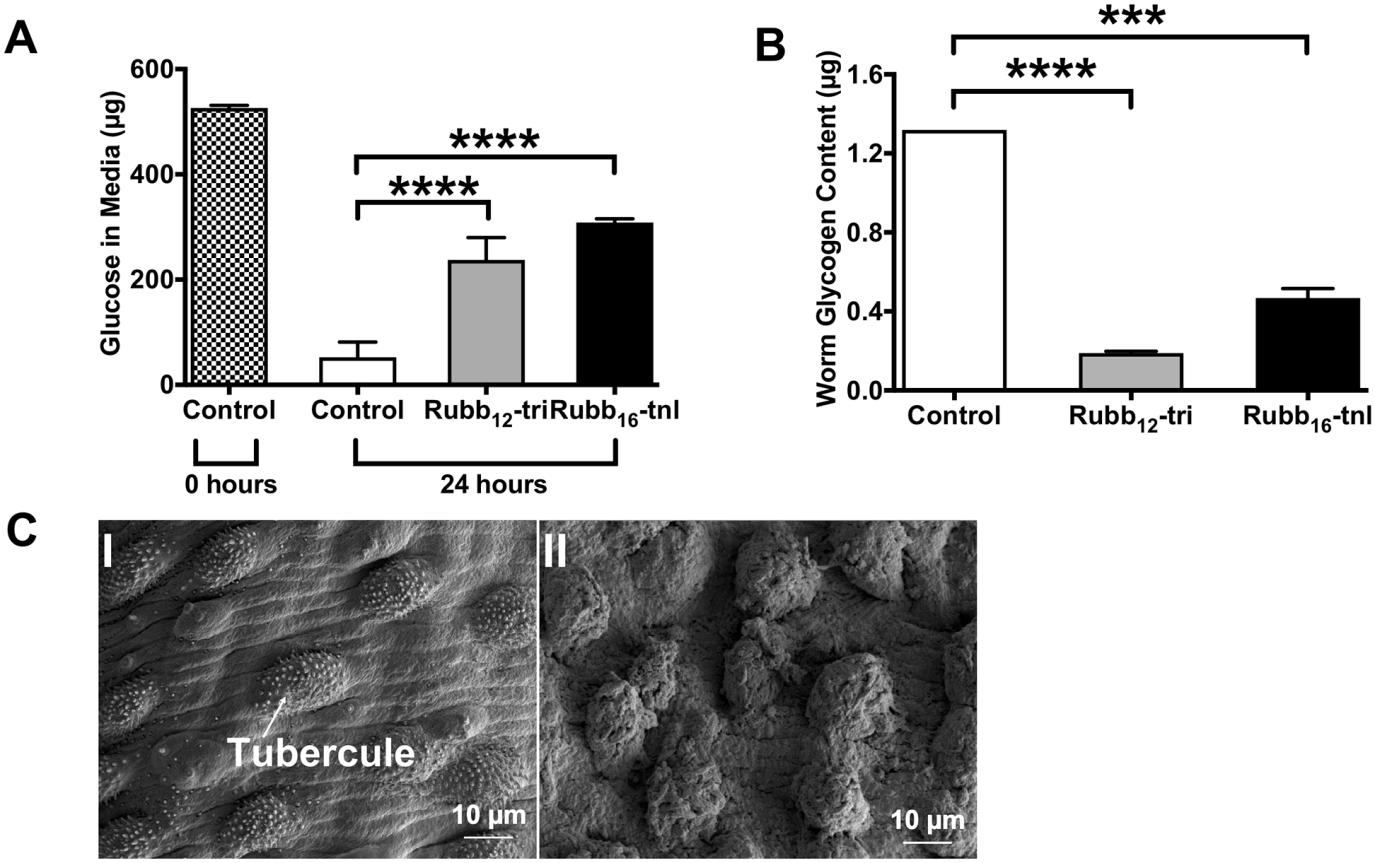
Effect of Rubb_12_-tri and Rubb_16_-tnl on adult *S*. *mansoni* glucose uptake and storage ability. Worms were cultured in Basch media for 24 h in the presence of a sub-lethal dose (5 μM) of Rubb_12_-tri or Rubb_16_-tnl. Worms were then incubated for 24 h in DMEM containing 1 mg/ml glucose. PBS extracts were then made from equal amounts of control and treated worms and 30 ug of each extract was used to determine the glycogen content of the worms using a modified glucose oxidase assay. (A) Amount of glucose in media collected from control and treated *S*. *mansoni* worms. (B) Levels of glycogen in extracts made from control and treated *S*. *mansoni* worms. For all assays, data are the average of triplicate biological and technical experiments ± SE. Differences were measured by ANOVA. *P ≤ 0.05, **P ≤ 0.01, ***P ≤ 0.001, **** *P* ≤ 0.0001. (C) Scanning electron micrographs of adult male *S*. *mansoni* worm tegument after incubation with 5 μM Rubb_12_–tri; (I) intact tubercles of control worms; (II) withered tubercules of treated worms.

The amount of glucose in the media of parasites treated with either complex was significantly higher than control worms (Fig 6A), suggestive of impaired glucose uptake in the presence of ruthenium compounds. Consistent with these results, extracts of treated parasites had a significantly lower glycogen content compared to controls (Fig 6B). Moreover, scanning electron micrographs of the tegument of male parasites treated with Rubb_12_-tri or Rubb_16_-tnl showed the dorsal tubercules (a site of glycogen storage [38]) to be withered and flattened (Fig 6C).

### Toxicity of Rubb_12_-tri and Rubb_7_-tnl

**Fig 7:**
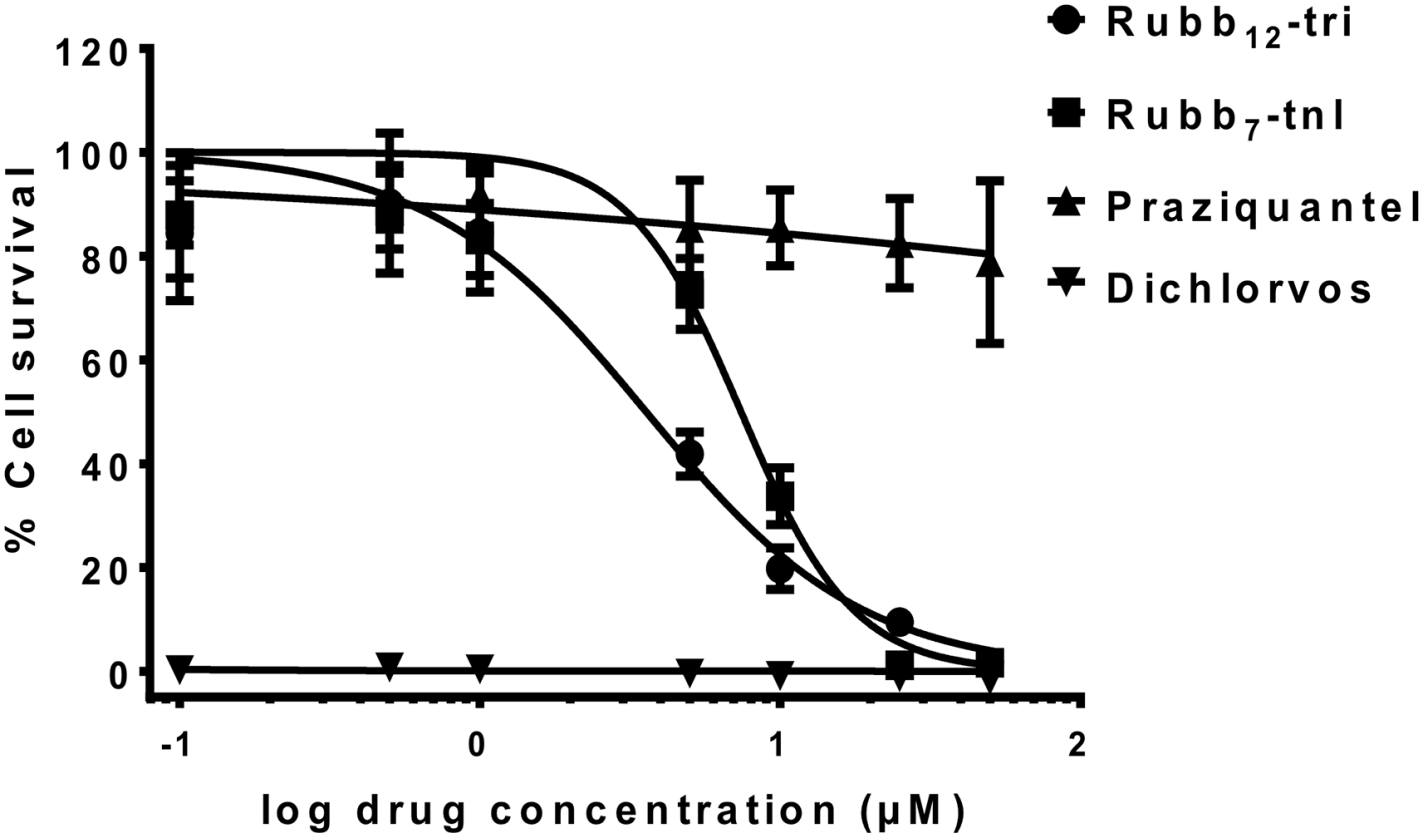
Cytotoxicity of ruthenium complexes. Toxicity against human bile duct cells (H69) after 72 h incubation with Rubb_12_-tri or Rubb_7_-tnl, praziquantel and dichlorvos—an organophosphorus AChE inhibitor—as determined by the MTT cell viability assay. Data are the average of six replicate experiments ± SE.

The toxicity of Rubb_12_-tri and Rubb_7_-tnl, two of the ruthenium complexes shown to have high *in vitro* efficacy against all schistosome stages tested, was assessed against human bile duct cells and in male BALB/c mice (6–8 weeks) before investigating their *in vivo* efficacy in a mouse model of schistosomiasis. PZQ and dichlorvos—a metabolite of the anti-schistosome AChE inhibitor metrifonate—were included in the study for comparison. When Rubb_12_-tri and Rubb_7_-tnl were used at 5 μM, a concentration where they significantly inhibited surface and somatic *Sm*-AChE activity and glucose uptake in adult worms, cell viability was 42% and 73% for Rubb_12_-tri and Rubb_7_-tnl, respectively. The EC_50_ values of Rubb_12_-tri and Rubb_7_-tnl were calculated as 3.489 ± 0.532 μM and 6.829 ± 0.625 μM, respectively. Dichlorvos was highly toxic to the cells, killing 100% of the cells even at 0.1 μM (Fig 7). To determine the MTD in mice, Rubb_12_-tri or Rubb_7_-tnl was administered to groups of male BALB/c mice (6–8 weeks). Rubb_7_-tnl did not show any toxicity even after five consecutive daily injections (the proposed dosage frequency of the *in vivo* drug efficacy study) of 10 mg/kg (mice were adversely affected at doses of 20 mg/kg) and so the MTD of Rubb_7_-tnl was considered to be at least 10 mg/kg. The MTD of Rubb_12_-tri, using the same dosage frequency, was determined to be 2 mg/kg (mice were adversely affected at doses of 4 mg/kg). If 100% bioavailability is assumed due to i.v. administration, the host bloodstream concentrations of Rubb_7_-tnl and Rubb_12_-tri at the MTD can be approximated at 12 μM and 49 μM, respectively.

### *In vivo* efficacy of Rubb_12_-tri and Rubb_7_-tnl

**Fig 8:**
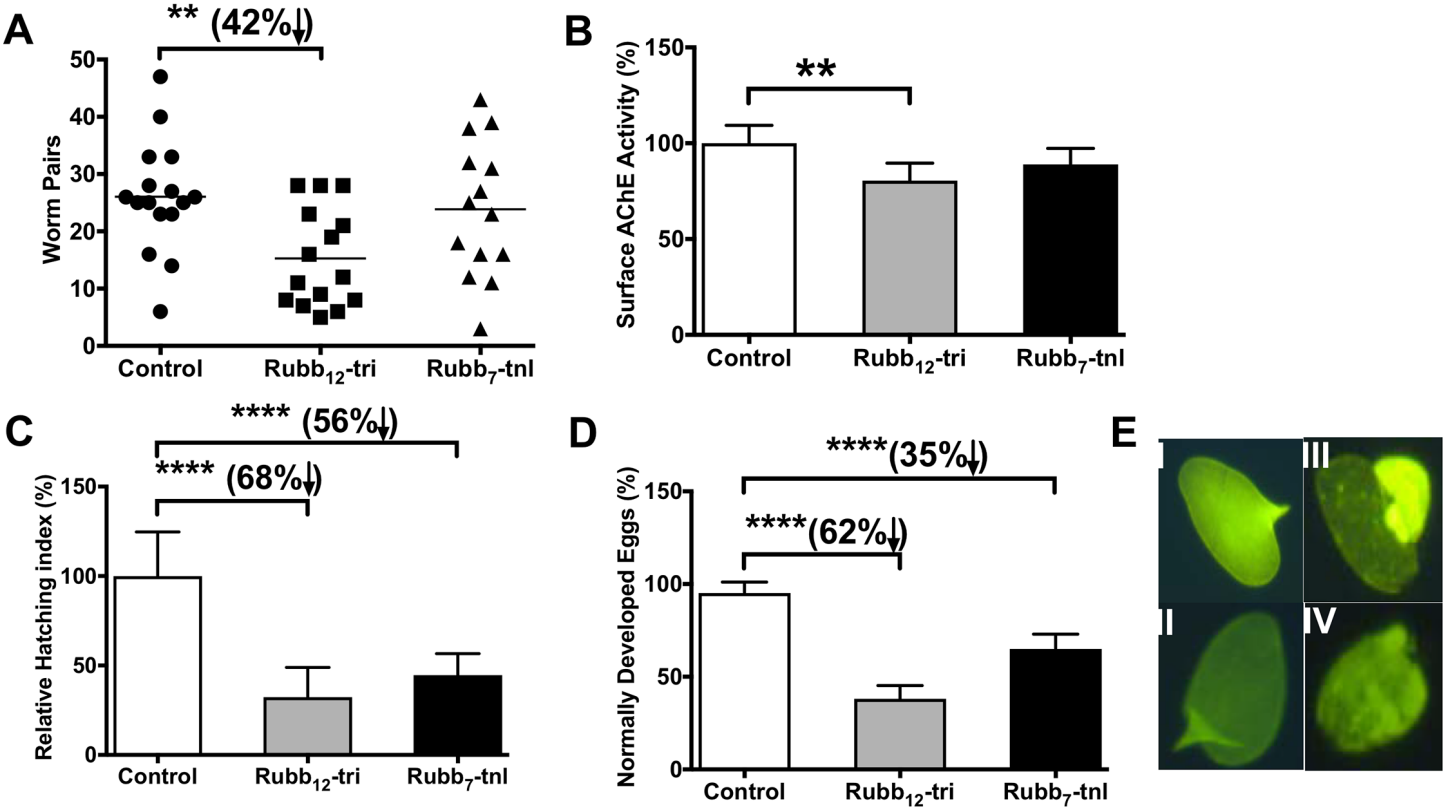
*In vivo* effect of Rubb_12_-tri and Rubb_7_-tnl on *S*. *mansoni*-infected mice. (A) Effect of Rubb_12_-tri and Rubb_7_-tnl on adult worm burden. Symbols represent data from individual mice and are the combination of two independent trials (trial 1 PBS control—n = 8 mice, trial 1 Rubb_12_-tri-treated—n = 8 mice, trial 1 Rubb_7_-tnl-treated—n = 6 mice, trial 2 PBS control—n = 7 mice, trial 2 Rubb_12_-tri-treated—n = 7 mice, trial 2 Rubb_7_-tnl-treated—n = 8 mice). (B) Surface AChE activity of worms recovered from control and treated mice. Data are the average of triplicate technical assays ± SE on extracts made from worms (five pairs) pooled from each group of each of the two trials. (C) Hatching viability of eggs obtained from the pooled livers of control and treated mice from trial 1 (PBS control—n = 8, Rubb_12_-tri-treated—n = 8, Rubb_7_-tnl-treated—n = 6). Data are the average of ten replicate counts ± SE of hatched miracidia. (D) Eggs were harvested from triplicate sets of worms (five pairs) from a pool of each group of trial 2 (PBS control—n = 7 mice, Rubb_12_-tri-treated—n = 7 mice, Rubb_7_-tnl-treated—n = 8 mice) after culturing the parasites for 24 h in Basch media and the percentage of mature, morphologically “normal” eggs released from worms recovered from control and treated mice was assessed. Data are the average counts ± SE of eggs released from triplicate sets (five pairs) of worms from trial 2. Differences were measured by ANOVA. *P ≤ 0.05, **P ≤ 0.01, ***P ≤ 0.001, **** *P* ≤ 0.0001. (E) Auto-fluorescence images (20×) of eggs released from worms recovered from (I and II) control and (III and IV) treated mice.

Over two independent trials, a significant reduction in worm burden (42%, *P* = 0.009) was seen in mice treated with Rubb_12_-tri compared to controls whereas a non-significant trend towards decreased worm burden was observed in Rubb_7_-tnl-treated mice (Fig 8A). Surface AChE activity was decreased in worms collected from mice treated with ruthenium complexes but only reached significance for Rubb_12_-tri-treated animals (Fig 8B). Surface SmNPP-5, surface *Sm*-AP, somatic AChE and glucose uptake activity was not significantly different.

Although there was no decrease in parasite egg burden (as determined by recovery of ova from the liver), the viability of these eggs from trial 1 was examined and highly significant (*P* < 0.0001) reductions in hatching capability (68% and 56%) were observed for the Rubb_12_-tri- and Rubb_7_-tnl-treated mice, respectively (Fig 8C). Egg hatching viability was not determined for trial 2. Moreover, eggs released from worms recovered from treated mice (trial 2) were significantly different (*P* < 0.0001) in terms of their development (immature, misshapen, eggshell malformation) compared to those from parasites recovered from control mice (Fig 8D and 8E). Morphology of eggs released from worms recovered from mice in trial 1 was not determined.

## Discussion

Control of schistosomiasis, a neglected tropical disease which affects over 200 million people, relies on periodic treatment with single drug, PZQ, a strategy that is unsustainable in its current form [5–8]. As no new anti-schistosome drug (or any anti-parasitic) has been registered in the last decade [39], the need for additional therapeutic compounds has become unquestionable and has driven research efforts towards the discovery of alternative anti-schistosome chemotherapies, including those derived from natural products [40] and metal-based compounds [41]. Accordingly, this study has described the anti-schistosome efficacy of a series of mono- and multi-nucleated metal-based compounds (ruthenium complexes) which exert their action through the inhibition of AChE, an enzyme pivotal to the control of worm neuromuscular function and implicated in the mediation of host glucose scavenging [11, 42].

It has been previously shown that mononuclear ruthenium complexes inhibit AChE (*E*. *electricus*) by a non-competitive or mixed mode of inhibition [18]. However, in the present study, the trinuclear complex (Rubb_12_-tri) displayed a competitive mode of inhibition in the kinetic experiments. The mononuclear polypyridylruthenium(II) complexes are thought to interact with the peripheral anionic site (PAS) of AChE located at the rim of the active-centre gorge through a combination of electrostatic and hydrophobic interactions [43]. The tri- and tetra-nuclear complexes showed greater activity compared to mononuclear complexes, presumably due to the presence of the flexibly-linked multiple metal centres which may provide more interactions (electrostatic and hydrophobic) with the PAS, or each individual centre may contribute nonspecific additional points of contact. The activity of the ruthenium complexes varied in extracts made from different life stages (adult extracts and SEA) and various species of the parasite which is most likely due to differences in enzyme orthologues and the existence of multiple isoforms of AChE which are present in different life stages and species [42].

Encouraged by the activity of ruthenium complexes against parasite extracts, we tested the compounds against all three intra-mammalian stages of the parasite *in vitro* and found similar trends in anti-parasite activity as was seen for extracts; i.e., the tri- and tetra-nucleated complexes were more effective against each stage of the parasite than the mono- and di-nuclear compounds. Three of the most effective compounds in terms of their combined activity against *S*. *mansoni* extracts and all intra-mammalian stages were Rubb_7_-tnl, Rubb_12_-tri and Rubb_16_-tnl. Further, Rubb_12_-tri was also the most effective at inhibiting AChE activity in *S*. *haematobium* extracts; availability of material prevented us from doing any experiments on live parasites or eggs but we believe that that the similar trends observed between *S*. *mansoni* anti-parasitic activity and extract activity hold true for *S*. *haematobium* and ruthenium complexes such as Rubb_12_-tri would display potent anti-schistosome activity against this species. Further, *S*. *haematobium* has higher levels of tegumental AChE than *S*. *mansoni*, which makes the parasite more sensitive to AChE inhibitors [44] and might render this species more vulnerable to ruthenium drugs.

Any differences observed between the AChE-inhibitory ability and anti-schistosome effect of ruthenium complexes could be due to the target of these drugs not solely being AChE. While we showed that ruthenium complexes did not have any inhibitory effects on the major tegumental enzymes SmNPP-5 and AP, these drugs have been documented to act as dual inhibitors of telomerase and topoisomerase [45], thioredoxin reductase [46] and protein and lipid kinases [43]. There are numerous reports in the literature documenting the development of drug resistance in parasites due to mutation (for example, benzamidazole resistance in nematodes due to single nucleotide polymorphisms in β-tubulin [47] and mutation of a schistosome sulfotransferase resulting in resistance to oxamniquine [48], and so the use of a drug that is directed against multiple molecular targets may decrease the chance of resistance evolving.

Visually, the effects of the compounds were most pronounced against adult worms, which became immobile and coiled when incubated with ruthenium complexes, possibly due to paralysis induced by AChE inhibition and cholinergic accumulation, effects similarly seen in schistosomes treated with other inhibitors of AChE [49–51]. This observation should be treated with caution, however, as other drugs, such as PZQ (which is not a cholinesterase inhibitor), induce the same morphological changes. Additional evidence of the mechanistic effects of ruthenium complexes on schistosomes manifested in the reduced glucose uptake observed in drug-treated worms, potentially a consequence of inhibiting the tegumental AChE-mediated regulation of host glucose scavenging, a pathway unique to schistosomes [11]. Further confirmation of this inhibition was evidenced by significantly depleted glycogen stores (quantified in parasite extracts and observed by the withering of male tubercules—a site of glycogen storage [38]) in these parasites, an effect seen in worms recovered from mice treated with carbamate-based AChE inhibitors [38]. In another example of tegument-mediated glucose regulation, previous work by You and colleagues [52] has shown that inhibition of schistosome insulin receptor activity significantly decreased glucose uptake by the parasite. It would be interesting to explore any relationship that existed between these two regulatory mechanisms, a possibility given the alternative is to imagine the evolution of two mechanistically distinct pathways of glucose regulation. Additionally, a combination chemotherapeutic strategy could be developed using drugs which target different aspects of schistosome glucose uptake.

Two of the ruthenium complexes which we considered most effective *in vitro* (Rubb_12_-tri and Rubb_7_-tnl) were tested for cytotoxicity before assessment of their *in vivo* efficacy in a mouse model of schistosomiasis. Rubb_16_-tnl, even though effective *in vitro*, was not included in the cytotoxicity assay or the *in vivo* study as earlier work by us has shown that ruthenium complexes with longer chain lengths are more toxic to eukaryotic cells [24]. Both ruthenium complexes tested, Rubb_7_-tnl and Rubb_12_-tri, exhibited lower cytotoxicity against eukaryotic cells (H69) compared to dichlorvos, an organophosphorus AChE inhibitor and previously licensed, but now withdrawn, anti-schistosome drug. Further, studies comparing AChE from schistosomes and higher eukaryotes [53, 54] reveal differences in functionally important amino acid residues (human AChE shares 33–36% primary sequence homology across all schistosome AChEs) with the active site serine conserved across species. It was previously shown that dichlorvos covalently binds to the active site serine and reduces the AChE activity in eukaryotes (e.g. in rat forebrain, erythrocytes and plasma) [55, 56]. By contrast, it was considered that the ruthenium complexes may be relatively less toxic to mice than dichlorvos due to the differential binding to AChE. The results of the MTD study, where both the ruthenium complexes were well tolerated by mice, supported this argument.

Rubb_12_-tri and Rubb_7_-tnl both showed promising *in vivo* efficacy at doses which were equivalent to lethal *in vitro* concentrations yet well tolerated in mice, with Rubb_12_-tri-treated mice showing a significant reduction in worm burden and recovered worms displaying a small but significant decrease in tegumental AChE activity, providing evidence that the anti-schistosome effect may be partially due to AChE inhibition. Studies by us on di-nuclear ruthenium complexes have shown the compounds to have a short serum half-life [57], and the different *in vivo* efficacies of each complex in this study may be attributed to differences in the pharmacokinetic/pharmacodynamic (PK/PD) properties of Rubb_12_-tri and Rubb_7_-tnl. Although these experiments have yet to be performed, the differences exhibited between these two compounds in the cytotoxicity assay and tolerability study suggests that their PK/PD are not the same. Despite no significant reduction in egg burden in both trials, ova recovered from both Rubb_12_-tri- and Rubb_7_-tnl-treated mice had significantly reduced hatching ability and were morphologically abnormal compared to controls, in agreement with *in vitro* data. That there was no decrease in egg burden in light of a reduced worm load in either treated group was surprising, but this did result in an increase in the number of eggs per female in these groups, compared to controls. To explain these observations, we postulate that treatment with ruthenium drugs may stimulate schistosome reproductive tract motility (AChE inhibitors have been shown to stimulate gastrointestinal motility in various organisms [58, 59]), resulting in premature release of under-developed eggs, and have a direct effect on egg formation, resulting in abnormally developed eggs (studies in ticks have shown that treatment with AChE inhibitors effect ova development [60, 61]). Another possible factor contributing to abnormal egg development is that the worms are under-nourished due an impaired glucose uptake ability (albeit an effect we could only measure *in vitro*) resulting from ruthenium drug treatment and unable to meet the energetically demanding task of producing normally developed ova. There is considerable interest in the use of agents that show ovicidal activity or affect oviposition to control schistosomiasis due to their ability to block transmission of the disease. In this regard, ruthenium complexes offer a potential advantage over PZQ in that it is only effective against mature worms and so cannot be used to interrupt disease transmission, as evidenced by high rates of re-infection in PZQ-treated endemic populations [62].

To our knowledge, this is the first report detailing the anti-parasitic activity of ruthenium complexes and this work has identified some lead anti-schistosome compounds. The modular nature of ruthenium complexes makes it possible to synthesize these compounds to target specific enzymes, so future work will involve tailoring Ru complexes to increase their selectivity and potency. Finally, these complexes could be administered in combination with PZQ, overcoming the limitations of current monotherapy and augmenting existing schistosomiasis control initiatives.

## Supporting information

S1 Table Inhibition of nucleotide pyrophosphatase-phosphodiesterase 5 (SmNPP-5) and alkaline phosphatase (AP) activity in adult *S*. *mansoni* Triton X-100-soluble extracts by a series of ruthenium complexes. (DOCX)

## References

[1] Gryseels B, Polman K, Clerinx J, Kestens L. Human schistosomiasis. Lancet. 2006;368(9541):1106–18. doi: 10.1016/S0140-6736(06)69440-3 . 16997665 10.1016/S0140-6736(06)69440-3

[2] Hotez PJ, Bethony JM, Diemert DJ, Pearson M, Loukas A. Developing vaccines to combat hookworm infection and intestinal schistosomiasis. Nat Rev Microbiol. 2010;8(11):814–26. Epub 2010/10/16. doi: 10.1038/nrmicro2438 . 20948553 10.1038/nrmicro2438

[3] Hams E, Aviello G, Fallon PG. The schistosoma granuloma: friend or foe? Frontiers in immunology. 2013;4:89 doi: 10.3389/fimmu.2013.00089 23596444 10.3389/fimmu.2013.00089 PMC3625856

[4] Molehin AJ, Rojo JU, Siddiqui SZ, Gray SA, Carter D, Siddiqui AA. Development of a schistosomiasis vaccine. Expert Rev Vaccines. 2016;15(5):619–27. doi: 10.1586/14760584.2016.1131127 26651503 10.1586/14760584.2016.1131127 PMC5070536

[5] Vale N, Gouveia MJ, Rinaldi G, Brindley PJ, Gartner F, Correia da Costa JM. Praziquantel for Schistosomiasis: Single-Drug Metabolism Revisited, Mode of Action, and Resistance. Antimicrob Agents Chemother. 2017;61(5). doi: 10.1128/AAC.02582-16 28264841 10.1128/AAC.02582-16 PMC5404606

[6] Seto EY, Wong BK, Lu D, Zhong B. Human schistosomiasis resistance to praziquantel in China: should we be worried? Am J Trop Med Hyg. 2011;85(1):74–82. doi: 10.4269/ajtmh.2011.10-0542 21734129 10.4269/ajtmh.2011.10-0542 PMC3122348

[7] Wang W, Wang L, Liang YS. Susceptibility or resistance of praziquantel in human schistosomiasis: a review. Parasitol Res. 2012;111(5):1871–7. doi: 10.1007/s00436-012-3151-z . 23052781 10.1007/s00436-012-3151-z

[8] Bergquist R, Utzinger J, Keiser J. Controlling schistosomiasis with praziquantel: How much longer without a viable alternative? Infect Dis Poverty. 2017;6(1):74 doi: 10.1186/s40249-017-0286-2 28351414 10.1186/s40249-017-0286-2 PMC5371198

[9] Massoulie J, Pezzementi L, Bon S, Krejci E, Vallette FM. Molecular and cellular biology of cholinesterases. Prog Neurobiol. 1993;41(1):31–91. . 8321908 10.1016/0301-0082(93)90040-y

[10] Tarrab-Hazdai R, Levi-Schaffer F, Smolarsky M, Arnon R. Acetylcholinesterase of Schistosoma mansoni: antigenic cross-reactivity with Electrophorus electricus and its functional implications. Eur J Immunol. 1984;14(3):205–9. doi: 10.1002/eji.1830140302 . 6705822 10.1002/eji.1830140302

[11] Camacho M, Agnew A. Schistosoma: rate of glucose import is altered by acetylcholine interaction with tegumental acetylcholine receptors and acetylcholinesterase. Exp Parasitol. 1995;81(4):584–91. doi: 10.1006/expr.1995.1152 . 8543000 10.1006/expr.1995.1152

[12] Camacho M, Alsford S, Jones A, Agnew A. Nicotinic acetylcholine receptors on the surface of the blood fluke Schistosoma. Mol Biochem Parasitol. 1995;71(1):127–34. . 7630376 10.1016/0166-6851(94)00039-p

[13] Skelly PJ, Da’dara AA, Li XH, Castro-Borges W, Wilson RA. Schistosome feeding and regurgitation. PLoS Pathog. 2014;10(8):e1004246 doi: 10.1371/journal.ppat.1004246 25121497 10.1371/journal.ppat.1004246 PMC4133383

[14] Thapa S, Lv M, Xu H. Acetylcholinesterase: A Primary Target for Drugs and Insecticides. Mini Rev Med Chem. 2017 doi: 10.2174/1389557517666170120153930 . 28117022 10.2174/1389557517666170120153930

[15] Kwong TC. Organophosphate pesticides: biochemistry and clinical toxicology. Therapeutic drug monitoring. 2002;24(1):144–9. Epub 2002/01/24. . 11805735 10.1097/00007691-200202000-00022

[16] Orhan IE. Nature: a substantial source of auspicious substances with acetylcholinesterase inhibitory action. Curr Neuropharmacol. 2013;11(4):379–87. doi: 10.2174/1570159X11311040003 24381529 10.2174/1570159X11311040003 PMC3744902

[17] Kramer CV, Zhang F, Sinclair D, Olliaro PL. Drugs for treating urinary schistosomiasis. Cochrane Database Syst Rev. 2014;(8):CD000053 doi: 10.1002/14651858.CD000053.pub3 25099517 10.1002/14651858.CD000053.pub3 PMC4447116

[18] Vyas NA, Bhat SS, Kumbhar AS, Sonawane UB, Jani V, Joshi RR, et al Ruthenium(II) polypyridyl complex as inhibitor of acetylcholinesterase and Abeta aggregation. Eur J Med Chem. 2014;75:375–81. doi: 10.1016/j.ejmech.2014.01.052 . 24556150 10.1016/j.ejmech.2014.01.052

[19] Gorle AK, Feterl M, Warner JM, Wallace L, Keene FR, Collins JG. Tri- and tetra-nuclear polypyridyl ruthenium(II) complexes as antimicrobial agents. Dalton Trans. 2014;43(44):16713–25. doi: 10.1039/c4dt02139h . 25271478 10.1039/c4dt02139h

[20] Li F, Mulyana Y, Feterl M, Warner JM, Collins JG, Keene FR. The antimicrobial activity of inert oligonuclear polypyridylruthenium(II) complexes against pathogenic bacteria, including MRSA. Dalton Trans. 2011;40(18):5032–8. doi: 10.1039/c1dt10250h . 21442118 10.1039/c1dt10250h

[21] Pandrala M, Li F, Feterl M, Mulyana Y, Warner JM, Wallace L, et al Chlorido-containing ruthenium(II) and iridium(III) complexes as antimicrobial agents. Dalton Trans. 2013;42(13):4686–94. doi: 10.1039/c3dt32775b . 23360972 10.1039/c3dt32775b

[22] Bourne Y, Radic Z, Kolb HC, Sharpless KB, Taylor P, Marchot P. Structural insights into conformational flexibility at the peripheral site and within the active center gorge of AChE. Chem Biol Interact. 2005;157–158:159–65. doi: 10.1016/j.cbi.2005.10.018 . 16259971 10.1016/j.cbi.2005.10.018

[23] Mason AJ, Marquette A, Bechinger B. Zwitterionic phospholipids and sterols modulate antimicrobial peptide-induced membrane destabilization. Biophys J. 2007;93(12):4289–99. doi: 10.1529/biophysj.107.116681 17766347 10.1529/biophysj.107.116681 PMC2098721

[24] Gorle AK, Li X, Primrose S, Li F, Feterl M, Kinobe RT, et al Oligonuclear polypyridylruthenium(II) complexes: selectivity between bacteria and eukaryotic cells. J Antimicrob Chemother. 2016;71(6):1547–55. doi: 10.1093/jac/dkw026 . 26945708 10.1093/jac/dkw026

[25] Lewis FA, Stirewalt MA, Souza CP, Gazzinelli G. Large-scale laboratory maintenance of Schistosoma mansoni, with observations on three schistosome/snail host combinations. J Parasitol. 1986;72(6):813–29. . 3546654

[26] Dalton JP, Day SR, Drew AC, Brindley PJ. A method for the isolation of schistosome eggs and miracidia free of contaminating host tissues. Parasitology. 1997;115 (Pt 1):29–32. . 9226954 10.1017/s0031182097001091

[27] Tucker MS, Karunaratne LB, Lewis FA, Frietas TC, Liang Y-S. Schistosomiasis In: Coico R, editor. Current Protocols in Immunology: John Wiley and Sons, Inc; 2013 p. 19.1.1–.1.57. 10.1002/0471142735.im1901s103 24510597

[28] Ellman GL, Courtney KD, Andres V, Featherstone RM. A new and rapid colorimetric determination of acetylcholinesterase activity. Biochemical Pharmacology. 1961;7(2):88–95. http://dx.doi.org/10.1016/0006-2952(61)90145-9. 13726518 10.1016/0006-2952(61)90145-9

[29] Rofatto HK, Tararam CA, Borges WC, Wilson RA, Leite LC, Farias LP. Characterization of phosphodiesterase-5 as a surface protein in the tegument of Schistosoma mansoni. Mol Biochem Parasitol. 2009;166(1):32–41. doi: 10.1016/j.molbiopara.2009.02.006 . 19428670 10.1016/j.molbiopara.2009.02.006

[30] Cesari IM, Simpson AJ, Evans WH. Properties of a series of tegumental membrane-bound phosphohydrolase activities of Schistosoma mansoni. Biochem J. 1981;198(3):467–73. 6275849 10.1042/bj1980467 PMC1163290

[31] Wangchuk P, Pearson MS, Giacomin PR, Becker L, Sotillo J, Pickering D, et al Compounds Derived from the Bhutanese Daisy, Ajania nubigena, Demonstrate Dual Anthelmintic Activity against Schistosoma mansoni and Trichuris muris. PLOS Neglected Tropical Diseases. 2016;10(8):e0004908 doi: 10.1371/journal.pntd.0004908 27490394 10.1371/journal.pntd.0004908 PMC4973903

[32] Rinaldi G, Loukas A, Brindley PJ, Irelan JT, Smout MJ. Viability of developmental stages of Schistosoma mansoni quantified with xCELLigence worm real-time motility assay (xWORM). Int J Parasitol Drugs Drug Resist. 2015;5(3):141–8. doi: 10.1016/j.ijpddr.2015.07.002 26288742 10.1016/j.ijpddr.2015.07.002 PMC4534758

[33] Pellegrino J, Oliveira CA, Faria J, Cunha AS. New approach to the screening of drugs in experimental schistosomiasis mansoni in mice. Am J Trop Med Hyg. 1962;11:201–15. . 14484966 10.4269/ajtmh.1962.11.201

[34] Gómez-Lechón MJ, Ponsoda X, Castell JV. A Microassay for Measuring Glycogen in 96-Well-Cultured Cells. Analytical Biochemistry. 1996;236(2):296–301. http://dx.doi.org/10.1006/abio.1996.0170. 8660508 10.1006/abio.1996.0170

[35] Pandrala M, Sundaraneedi MK, Ammit AJ, Woodward CE, Wallace L, Keene FR, et al Differential Anticancer Activities of the Geometric Isomers of Dinuclear Iridium(III) Complexes. European Journal of Inorganic Chemistry. 2015;2015(34):5694–701. doi: 10.1002/ejic.201501069

[36] Smout MJ, Sotillo J, Laha T, Papatpremsiri A, Rinaldi G, Pimenta RN, et al Carcinogenic Parasite Secretes Growth Factor That Accelerates Wound Healing and Potentially Promotes Neoplasia. PLOS Pathogens. 2015;11(10):e1005209 doi: 10.1371/journal.ppat.1005209 26485648 10.1371/journal.ppat.1005209 PMC4618121

[37] Tran MH, Pearson MS, Bethony JM, Smyth DJ, Jones MK, Duke M, et al Tetraspanins on the surface of Schistosoma mansoni are protective antigens against schistosomiasis. Nat Med. 2006;12(7):835–40. Epub 2006/06/20. doi: 10.1038/nm1430 . 16783371 10.1038/nm1430

[38] Bueding E, Schiller EL, Bourgeois JG. Some physiological, biochemical, and morphologic effects of tris (p-aminophenyl) carbonium salts (TAC) on Schistosoma mansoni. Am J Trop Med Hyg. 1967;16(4):500–15. . 4952150 10.4269/ajtmh.1967.16.500

[39] Pedrique B, Strub-Wourgaft N, Some C, Olliaro P, Trouiller P, Ford N, et al The drug and vaccine landscape for neglected diseases (2000–11): a systematic assessment. Lancet Glob Health. 2013;1(6):e371–9. doi: 10.1016/S2214-109X(13)70078-0 . 25104602 10.1016/S2214-109X(13)70078-0

[40] Wangchuk P, Pearson MS, Giacomin PR, Becker L, Sotillo J, Pickering D, et al Compounds Derived from the Bhutanese Daisy, Ajania nubigena, Demonstrate Dual Anthelmintic Activity against Schistosoma mansoni and Trichuris muris. PLoS Negl Trop Dis. 2016;10(8):e0004908 doi: 10.1371/journal.pntd.0004908 27490394 10.1371/journal.pntd.0004908 PMC4973903

[41] Kuntz AN, Davioud-Charvet E, Sayed AA, Califf LL, Dessolin J, Arner ES, et al Thioredoxin glutathione reductase from Schistosoma mansoni: an essential parasite enzyme and a key drug target. PLoS Med. 2007;4(6):e206 doi: 10.1371/journal.pmed.0040206 17579510 10.1371/journal.pmed.0040206 PMC1892040

[42] Arnon R, Silman I, Tarrab-Hazdai R. Acetylcholinesterase of Schistosoma mansoni—functional correlates. Contributed in honor of Professor Hans Neurath’s 90th birthday. Protein Sci. 1999;8(12):2553–61. doi: 10.1110/ps.8.12.2553 10631970 10.1110/ps.8.12.2553 PMC2144239

[43] Meggers E. Targeting proteins with metal complexes. Chem Commun (Camb). 2009;(9):1001–10. doi: 10.1039/b813568a . 19225621 10.1039/b813568a

[44] Camacho M, Tarrab-Hazdai R, Espinoza B, Arnon R, Agnew A. The amount of acetylcholinesterase on the parasite surface reflects the differential sensitivity of schistosome species to metrifonate. Parasitology. 1994;108 (Pt 2):153–60. . 8159460 10.1017/s0031182000068244

[45] Liao G, Chen X, Wu J, Qian C, Wang Y, Ji L, et al Ruthenium(II) polypyridyl complexes as dual inhibitors of telomerase and topoisomerase. Dalton Trans. 2015;44(34):15145–56. doi: 10.1039/c4dt03585b . 25604798 10.1039/c4dt03585b

[46] Luo Z, Yu L, Yang F, Zhao Z, Yu B, Lai H, et al Ruthenium polypyridyl complexes as inducer of ROS-mediated apoptosis in cancer cells by targeting thioredoxin reductase. Metallomics. 2014;6(8):1480–90. doi: 10.1039/c4mt00044g . 24823440 10.1039/c4mt00044g

[47] Von Samson-Himmelstjerna G, Blackhall WJ, McCarthy JS, Skuce PJ. Single nucleotide polymorphism (SNP) markers for benzimidazole resistance in veterinary nematodes. Parasitology. 2007;134(Pt 8):1077–86. doi: 10.1017/S0031182007000054 . 17608967 10.1017/S0031182007000054

[48] Valentim CL, Cioli D, Chevalier FD, Cao X, Taylor AB, Holloway SP, et al Genetic and molecular basis of drug resistance and species-specific drug action in schistosome parasites. Science. 2013;342(6164):1385–9. doi: 10.1126/science.1243106 24263136 10.1126/science.1243106 PMC4136436

[49] Bueding E, Liu CL, Rogers SH. Inhibition by metrifonate and dichlorvos of cholinesterases in schistosomes. Br J Pharmacol. 1972;46(3):480–7. 4656609 10.1111/j.1476-5381.1972.tb08145.x PMC1666567

[50] Hillman GR, Senft AW. Anticholinergic properties of the antischistosomal drug hycanthone. Am J Trop Med Hyg. 1975;24(5):827–34. . 1190369 10.4269/ajtmh.1975.24.827

[51] Pax RA, Siefker C, Hickox T, Bennett JL. Schistosoma mansoni: neurotransmitters, longitudinal musculature and effects of electrical stimulation. Exp Parasitol. 1981;52(3):346–55. . 6119224 10.1016/0014-4894(81)90092-8

[52] You H, Zhang W, Jones MK, Gobert GN, Mulvenna J, Rees G, et al Cloning and characterisation of Schistosoma japonicum insulin receptors. PLoS One. 2010;5(3):e9868 doi: 10.1371/journal.pone.0009868 . 20352052 10.1371/journal.pone.0009868 PMC2844434

[53] Bentley GN, Jones AK, Agnew A. Mapping and sequencing of acetylcholinesterase genes from the platyhelminth blood fluke Schistosoma. Gene. 2003;314:103–12. . 14527722 10.1016/s0378-1119(03)00709-1

[54] Jones AK, Bentley GN, Oliveros Parra WG, Agnew A. Molecular characterization of an acetylcholinesterase implicated in the regulation of glucose scavenging by the parasite Schistosoma. FASEB J. 2002;16(3):441–3. doi: 10.1096/fj.01-0683fje . 11821256 10.1096/fj.01-0683fje

[55] Hinz V, Grewig S, Schmidt BH. Metrifonate and dichlorvos: effects of a single oral administration on cholinesterase activity in rat brain and blood. Neurochem Res. 1996;21(3):339–45. . 9139240 10.1007/BF02531650

[56] Jann MW. Preclinical pharmacology of metrifonate. Pharmacotherapy. 1998;18(2 Pt 2):55–67; discussion 79–82. . 9543466

[57] Li F, Gorle AK, Ranson M, Vine KL, Kinobe R, Feterl M, et al Probing the pharmacokinetics of cucurbit[7, 8 and 10]uril: and a dinuclear ruthenium antimicrobial complex encapsulated in cucurbit[10]uril. Org Biomol Chem. 2017;15(19):4172–9. doi: 10.1039/c7ob00724h . 28443914 10.1039/c7ob00724h

[58] De Giorgio R, Stanghellini V, Barbara G, Guerrini S, Lioce A, Vasina V, et al Prokinetics in the treatment of acute intestinal pseudo-obstruction. IDrugs. 2004;7(2):160–5. . 15057661

[59] McNamara R, Mihalakis MJ. Acute colonic pseudo-obstruction: rapid correction with neostigmine in the emergency department. J Emerg Med. 2008;35(2):167–70. doi: 10.1016/j.jemermed.2007.06.043 . 18242923 10.1016/j.jemermed.2007.06.043

[60] Perez-Gonzalez IE, Prado-Ochoa MG, Munoz-Guzman MA, Vazquez-Valadez VH, Velazquez-Sanchez AM, Avila-Suarez BL, et al Effect of new ethyl and methyl carbamates on Rhipicephalus microplus larvae and adult ticks resistant to conventional ixodicides. Vet Parasitol. 2014;199(3–4):235–41. doi: 10.1016/j.vetpar.2013.07.042 . 24315692 10.1016/j.vetpar.2013.07.042

[61] Prado-Ochoa MG, Ramirez-Noguera P, Diaz-Torres R, Garrido-Farina GI, Vazquez-Valadez VH, Velazquez-Sanchez AM, et al The action of two ethyl carbamates on acetylcholinesterase and reproductive organs of Rhipicephalus microplus. Vet Parasitol. 2014;199(3–4):215–24. doi: 10.1016/j.vetpar.2013.10.028 . 24315691 10.1016/j.vetpar.2013.10.028

[62] Webster BL, Diaw OT, Seye MM, Faye DS, Stothard JR, Sousa-Figueiredo JC, et al Praziquantel treatment of school children from single and mixed infection foci of intestinal and urogenital schistosomiasis along the Senegal River Basin: monitoring treatment success and re-infection patterns. Acta Trop. 2013;128(2):292–302. doi: 10.1016/j.actatropica.2012.09.010 . 23022016 10.1016/j.actatropica.2012.09.010

