# Supplementary material for: Polypyridylruthenium(II) complexes exert anti-schistosome activity and inhibit parasite acetylcholinesterases
Source: PLoS Negl Trop Dis. 2017 Dec 14;11(12):e0006134. doi: 10.1371/journal.pntd.0006134 (PMC5746282; doi:10.1371/journal.pntd.0006134)
Supplement: S1 Table — (DOCX) [file pntd.0006134.s001.docx]

**S1 Table. Inhibition of nucleotide pyrophosphatase-phosphodiesterase 5 (SmNPP-5) and alkaline phosphatase (AP) activity in adult *S. mansoni* Triton X-100-soluble extracts by a series of ruthenium complexes.**

| **Compound** | **SmNPP-5 Inhibition (%)^a^** | **AP Inhibition (%)^a^** |
| --- | --- | --- |
| Ru(phen)_2_(Me_2_bpy) | 2 | 3 |
| Rubb_12_-mono | 15 | 2 |
| Rubb_12_-di | 1 | 1 |
| Rubb_7_-tri | 9 | 1 |
| Rubb_10_-tri | 12 | 8 |
| Rubb_12_-tri | 2 | 4 |
| Rubb_16_-tri | 15 | 6 |
| Rubb_7_-tl | 13 | 2 |
| Rubb_12_-tl | 3 | 2 |
| Rubb_16_-tl | 26 | 1 |
| Rubb_7_-tnl | 13 | 1 |
| Rubb_12_-tnl | 24 | 1 |

^a^inhibition of enzyme activites assessed at 10 µM.
